# Low concentrations of acetamiprid, deltamethrin, and sulfoxaflor, three commonly used insecticides, adversely affect ant queen survival and egg laying

**DOI:** 10.1038/s41598-023-42129-7

**Published:** 2023-09-09

**Authors:** Jakub Svoboda, Pavel Pech, Petr Heneberg

**Affiliations:** 1 Faculty of Science, University of Hradec Králové, Hradec Králové, Czech Republic; 2 Research and Breeding Institute of Pomology Holovousy Ltd., 508 01 Holovousy 129, Czech Republic; 3 Third Faculty of Medicine, Charles University, Ruská 87, 100 00 Prague, Czech Republic

**Keywords:** Entomology, Agroecology

## Abstract

Ants are key ecosystem service providers and can serve as important biological control agents in pest management. However, the effects of insecticides on common farmland ant species are poorly understood. We tested the effects of three commonly used insecticides on ants (Hymenoptera, Formicidae). The tested insecticides were acetamiprid (neonicotinoid; formulated as Mospilan 20 SP), deltamethrin (pyrethroid; formulated as Sanium Ultra), and sulfoxaflor (sulfilimine; formulated as Gondola). We tested two ant (Hymenoptera: Formicidae) species with different colony founding strategies, *Lasius niger* (Linnaeus, 1758) and *Myrmica rubra* (Linnaeus, 1758). We sprayed their queens with insecticides at concentrations recommended for use in foliar applications in agriculture, i.e., at 1.25 g L^−1^ (acetamiprid), 0.6 g L^−1^ (sulfoxaflor), and 0.875 g L^−1^ (deltamethrin). Further, we diluted the compounds in distilled water and tested them at 10%, 1%, and 0.1% of the field-recommended concentrations, and used distilled water as a control. We monitored the survival of the queens and the number of eggs laid. All three tested insecticides caused severe lethal and sublethal concentration-dependent effects. Even at concentrations three orders of magnitudes lower than recommended for field applications, significantly lower numbers of eggs were found in the queens’ nests. The extent of the sublethal effects of acetamiprid and sulfoxaflor was concentration-dependent and differed between the two ant species. Besides bees and bumblebees, ants represent an important group of hymenopterans that are severely affected even by low concentrations of the tested compounds and therefore should be included in risk assessment schemes.

## Introduction

The ideal insecticide should show high efficacy to target and low toxicity to non-target organisms. Unfortunately, it is seldom possible. The effects of low, sublethal concentrations of insecticides on non-target organisms are particularly important because these effects are difficult to observe until the changes in whole communities in nature occur. The spectrum of mandatorily tested invertebrate organisms is relatively narrow, and the mandatory tests focus predominantly on lethal effects and other easy-to-test effects^1,2^. Sublethal effects of insecticides in non-target organisms include changes in fertility, behavior, and interspecific interactions, including effects on insect parasitoids^3–6^. This leads to the disruption of ecosystem services provided by non-target organisms [e.g., Refs.^7–11^]. Ants represent key ecosystem service providers^12^. They have a great potential to serve as biological control agents^13–15^. Ants are essential in terrestrial ecosystems as predators, herbivores, scavengers, and seed dispersers^16^. Moreover, they strongly influence soil chemical and physical properties^17^.

The key to the high fitness of these eusocial insects is the survival and fertility of queens. For most of their life, the queens may be less susceptible to agrochemicals than workers. Data suggest that queens have a superior detoxification mechanisms compared to workers^18^, are hidden in the nests, and are therefore protected from direct exposure to freshly applied agrochemicals. After spraying droplets of insecticides on target plants, up to 30% of the compounds applied flow down from the plants to the soil surface^19^. Soil contamination can also occur by washing pesticides from the plant surface with water due to rain, dew, transpiration, or gutting of the plant^20^. Many ant species build their nests only a few centimeters below the soil surface^21^, and thereby they might be exposed to significant concentrations of applied insecticides. Moreover, queens may be exposed to agrochemicals during and after nuptial flights and searching of nest sites and may be chronically exposed to agrochemicals present in water and provisioned food.

Here, we focus on the effects of representatives of three groups of commonly used insecticides: neonicotinoids, pyrethroids, and sulfilimines. Neonicotinoids are widely used as a replacement for organophosphates and carbamates. Neonicotinoids are superior to them regarding the presence of only limited adverse effects on vertebrates^22^. Despite that, the negative impact of neonicotinoids on pollinators^7^ led to a ban on several neonicotinoids in many countries^23^. The neonicotinoids are highly mobile due to their solubility in water; therefore, they can enter soil water and remain there for up to two years following their application^20^. The ban on several neonicotinoid compounds led to their replacement with other agrochemicals^24,25^. Pyrethroids and sulfilimines predated neonicotinoids by over two decades. Despite many were displaced from the market, some are still allowed and broadly used. Pyrethroids induce insects' paralysis and death^26,27^. In contrast to neonicotinoids, pyrethroids are nonpolar and readily adsorb on soil and other particulate matter [e.g., Ref.^28^]. Ant queens can be typically exposed to pyrethroids by ingesting contaminated food; therefore, queens of ants with claustral colony founding mode may be protected against their effects. Sulfilimines have a similar mechanism of action as neonicotinoids but do not show cross-resistance^29^ (cross-resistance refers to the situation where the contact of an organism with a first compound confers changes that reduce the efficacy of a second, unrelated compound that may be in contact with the respective organism at a later time). The sulfoximine insecticide Gondola is already known to adversely affect the reproduction of bumblebees^30–32^. In contrast to the first generations of neonicotinoids, it does not have the anti-olfactory effects^33^. Sulfoxaflor, the active ingredient of Gondola, is also toxic to ants (Hymenoptera: Formicidae) *Solenopsis invicta* Buren, 1972 at 1–2 mg L^−1^ *p.o.*^34^ and *Tetramorium caespitum* (Linnaeus, 1758) at 1 mg L^−1^ *p.o.*^35^. Sulfoxaflor is still broadly used in many countries. Controversies regarding its effects resulted in long-lasting disputes between U.S. Environmental Protection Agency and the U.S. 9th Circuit Court of Appeals [e.g., Ref.^36^], and France terminated the registration of two sulfoxaflor formulations, Closer and Transform, in 2017^37^.

The present study aimed to elucidate the effects of commercial formulations of the neonicotinoid acetamiprid (formulated as Mospilan 20 SP), the pyrethroid deltamethrin (formulated as Sanium Ultra), and the sulfilimine sulfoxaflor (formulated as Gondola). All three have detrimental effects on soil organisms, such as earthworms^38–40^. Acetamiprid has negligible sorption and low mineralization rates; therefore, acetamiprid residues have extremely long persistence within the environment^41^. Deltamethrin also undergoes negligible mineralization and persists long in the environment^42,43^. Only sulfoxaflor has a short half-life in the soil (less than one day^44^), but it does not adsorb to solid particles and, therefore, can quickly disperse with the seeping water^45^. We tested the effects of the three insecticides on the survival and reproduction of queens of two ant (Hymenoptera: Formicidae) species that differ in colony founding strategies. As model species, we used the black garden ant *Lasius niger* (Linnaeus, 1758) and the European fire ant *Myrmica rubra* (Linnaeus, 1758). Different colony founding strategies (the need for food of sufficient quality and quantity during colony founding for species using semiclaustral colony founding) seem to stay behind a part of differences in species richness and diversity of ant communities in agrocenoses differing in the management type and intensity and the use of agrochemicals^46,47^. Based on our previous experiments with neonicotinoids and the previously reported data on the adverse effects of Gondola on the reproduction of bumblebees^30–32^, we hypothesized that sublethal concentrations of the tested insecticides affect the reproduction of ant queens. The two tested ant species differ in their colony founding strategies and thus have different sources of building blocks for their metabolism during the colony founding period^21^. Therefore, we further hypothesized that the effects of tested insecticides differ between the two unrelated ant species.

## Materials and methods

### Studied species

We used queens of two common ant (Hymenoptera, Formicidae) species, *L. niger* and *M. rubra* as model organisms. These species are distributed across the Palearctic and have been repeatedly introduced in North America^21,48–50^. The ecological requirements of the studied species partially overlap^51,52^ and may also be exposed similarly. Both species are common and abundant in open landscapes, light forests, and human settlements, but differ in colony founding^21^.

We collected 182 *L. niger* queens using a sweeping net after their nuptial flight on 22. July 2021 in Pecka (50°28.80' N, E 15°36.50' E) and 124 M*. rubra* queens on 2. July 2021 by digging them out of their nests in Hradec Králové (50°11.28' N, E 15°36.50' E) and on 4.–7. July 2021 in Jaroměř (50°21.74' N, 15°55.28' E).

### Experimental design

We designed the experiment as an acute contact (topical; applied as a direct spray on the organism) exposure of individually placed ant queens. We randomly assigned 14 queens of *L. niger* or 17–18 queens of *M. rubra* to each treatment type. We applied the insecticides using the auto-load Potter Precision Laboratory Spray Tower (Burkard Scientific, Uxbridge, United Kingdom). Before the treatment, the ant queens were allowed 11–16 days (*M. rubra*) or 18 days (*L. niger*) for acclimation under laboratory conditions at 22 °C, natural day/night cycle, and 40–60% humidity. While applying the insecticides, the ant queens were placed individually in Petri dishes. After the application, the ant queens were removed to clean Petri dishes and maintained as specified below.

**Figure 1:**
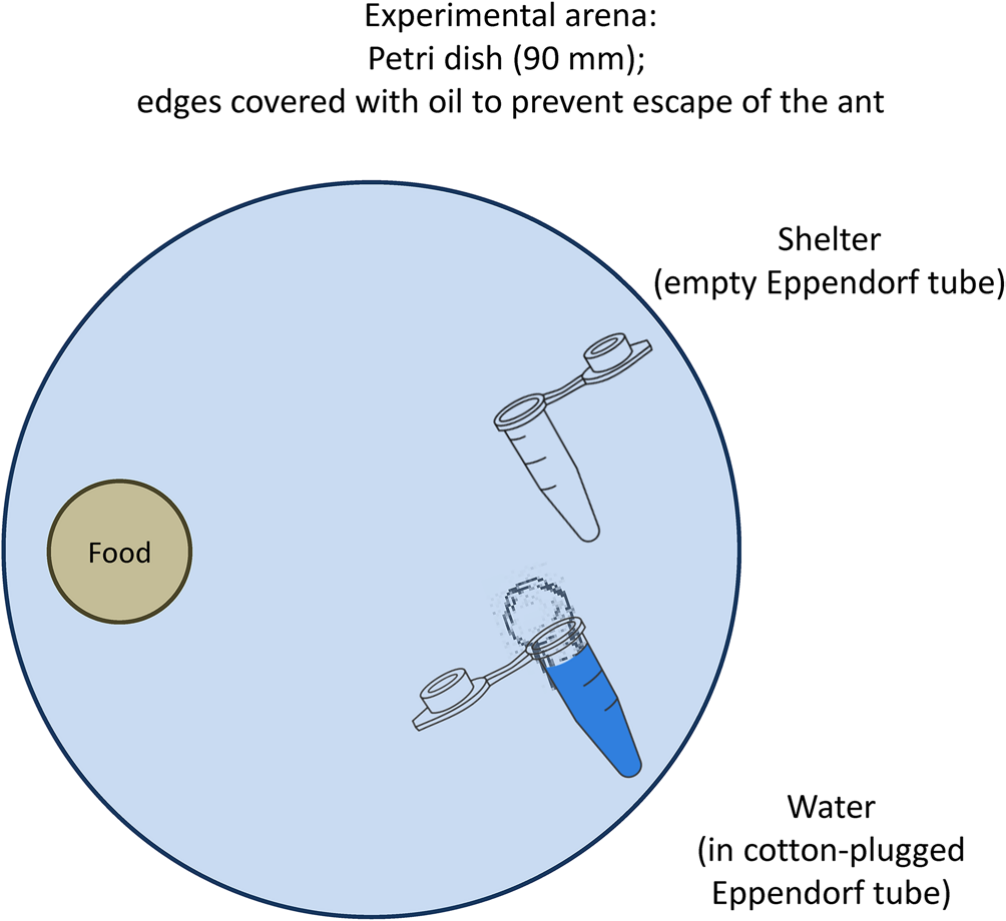
Design of experimental arenas to maintain the tested ant queens.

We kept the queens individually in polystyrene Petri dishes of 90 mm diameter under laboratory conditions at 22 °C, natural day/night cycle, and 40–60% humidity (Fig. 1). Each Petri dish was equipped with a plastic 1.5 ml Eppendorf tube filled with water and plugged by a piece of cotton wool and with another plastic 1.5 ml Eppendorf tube that was empty and served as a shelter. We supplemented the queens of *M. rubra* with one larva of *Tenebrio molitor* and a drop of honey once per three days. Queens of *L. niger* did not need to eat during the experiment as they represent a species with claustral colony founding. We terminated the experiment after six weeks, as soon as the first larva hatched, and counted the laid eggs immediately. We also measured the mortality of queens during the experiments, and calculated the mortality as the percentage of queens that died during the period from the start of the treatment until the termination of the experiment six weeks later. We monitored the survival every three days (during the feeding of *M. rubra*). Still, the exact time of the death of individual queens was not recorded except for those that died during the first 24 h following the administration of studied compounds.

### Insecticides

We exposed the ant queens to the following three insecticides: the neonicotinoid acetamiprid (formulated as Mospilan 20 SP; Nippon Soda Co. Ltd., Japan), the pyrethroid deltamethrin (formulated as Sanium Ultra; Dow AgroSciences s.r.o., Czech Republic), and the sulfilimine sulfoxaflor (formulated as Gondola; SBM Developpement S.A.S, France). These products are used as insecticides in foliar applications against herbivorous insect pests worldwide. Acetamiprid and sulfoxaflor are competitive inhibitors of nicotinic acetylcholine receptors, whereas deltamethrin acts as a phosphoprotein phosphatase inhibitor, a calcium channel agonist, and an antifeedant. According to the manufacturers' instructions, Mospilan 20 SP, which contains acetamiprid at 200 g kg^−1^, is recommended to be used at 250 g 200–1000 L^−1^ of H_2_O 10,000 m^−2^ in foliar application on fruit bushes and trees once to twice during the season^53^. The half-life of acetamiprid in the soil depends on moisture level and ranges between 16 and 151 days^54^. Sanium Ultra, which contains deltamethrin at 15 g L^−1^, is recommended to be used at 3.5 mL 4 L^−1^ of H_2_O 100 m^−2^ to treat potato fields^55^. Deltamethrin has low mobility in soil (but this does not apply to sandy soils^56^). Gondola, which contains sulfoxaflor at 120 g L^−1^, is recommended to be used at 200 mL 200–600 L of H_2_O 10,000 m^−2^ to treat potato fields^57^. Due to their physicochemical properties, Mospilan 20 SP and Gondola are distributed in the plants (and soil) systemically, whereas Sanium Ultra adsorbs only locally. Therefore, the application treatment with Sanium Ultra includes spraying the whole plant. As a control, we used distilled water.

As the recommended volumes per surface unit overlapped, we applied all three compounds in identical volumes (0.2544 mL 58 cm^−2^) (58 cm^2^ represents the surface area of a 90-mm Petri dish). We prepared the working concentrations of the tested insecticides, which corresponded to the concentrations recommended by the manufacturers for the use in foliar applications (further termed 100% concentrations): Mospilan 20 SP 1.25 g L^−1^, Gondola 0.6 g L^−1^, and Sanium Ultra 0.875 g L^−1^. We applied the working (100%) concentrations to the tested ants as specified below. Further, we diluted the working concentrations by 1:10 (further termed 10% concentrations), 1:100 (1% concentrations), and 1:1000 (0.1% concentrations). All four concentrations were used to treat queens of *L. niger*, while only the 100% and 10% concentrations were used for *M. rubra*. We initially used the 100% and 10% concentrations to treat *M. rubra*. To further extend the study and reflect the detrimental effects of the studied compounds in *M. rubra*, we next treated *L. niger* with 100%, 10%, 1%, and 0.1% concentrations of the studied compounds*.* Distilled water was used both as the vehicle and as a control.

### Statistics

Data are shown as the mean ± SE unless stated otherwise. As the obtained data were normally distributed (Shapiro–Wilk test p > 0.05) and had equal variance (Levene's test p > 0.05), we used one-way ANOVA with Bonferroni post-tests to compare the differences in effects of insecticides on the number of eggs produced by queens in *L. niger*. One-tailed *t*-test was used to compare the differences in effects of insecticides on the number of eggs produced by queens in *M. rubra*. To calculate LD_50_, we used Finney`s Probit Analysis^58^. To characterize the concentration dependence of the declines in the produced number of eggs, we performed polynomial regression analyses (linear regression for Mospilan and Gondola, and inverse third-order regression for Sanium Ultra). The analyses were performed in SigmaPlot 12.0.

## Results

### Mospilan

Among the three tested insecticides, Mospilan 20 SP was the only formulation that did not induce lethal effects on the tested queens of *L. niger* in any of the four concentrations, including the recommended concentration of 1.25 g L^−1^. The highest Mospilan concentration was associated with 14% mortality (2 out of 14 queens died). Among the queens treated with lower Mospilan concentrations (10%, 1%, and 0.1% of the field recommended concentrations), we recorded 7% mortality (1 out of 14 queens died in each treatment). This is the same mortality as in control, water-treated queens (7% mortality, 1 out of 14 control queens died). Because of limited Mospilan-induced mortality, the LD_50_ of Mospilan cannot be calculated (only a single point is available for Finney`s Probit Analysis).

**Figure 2:**
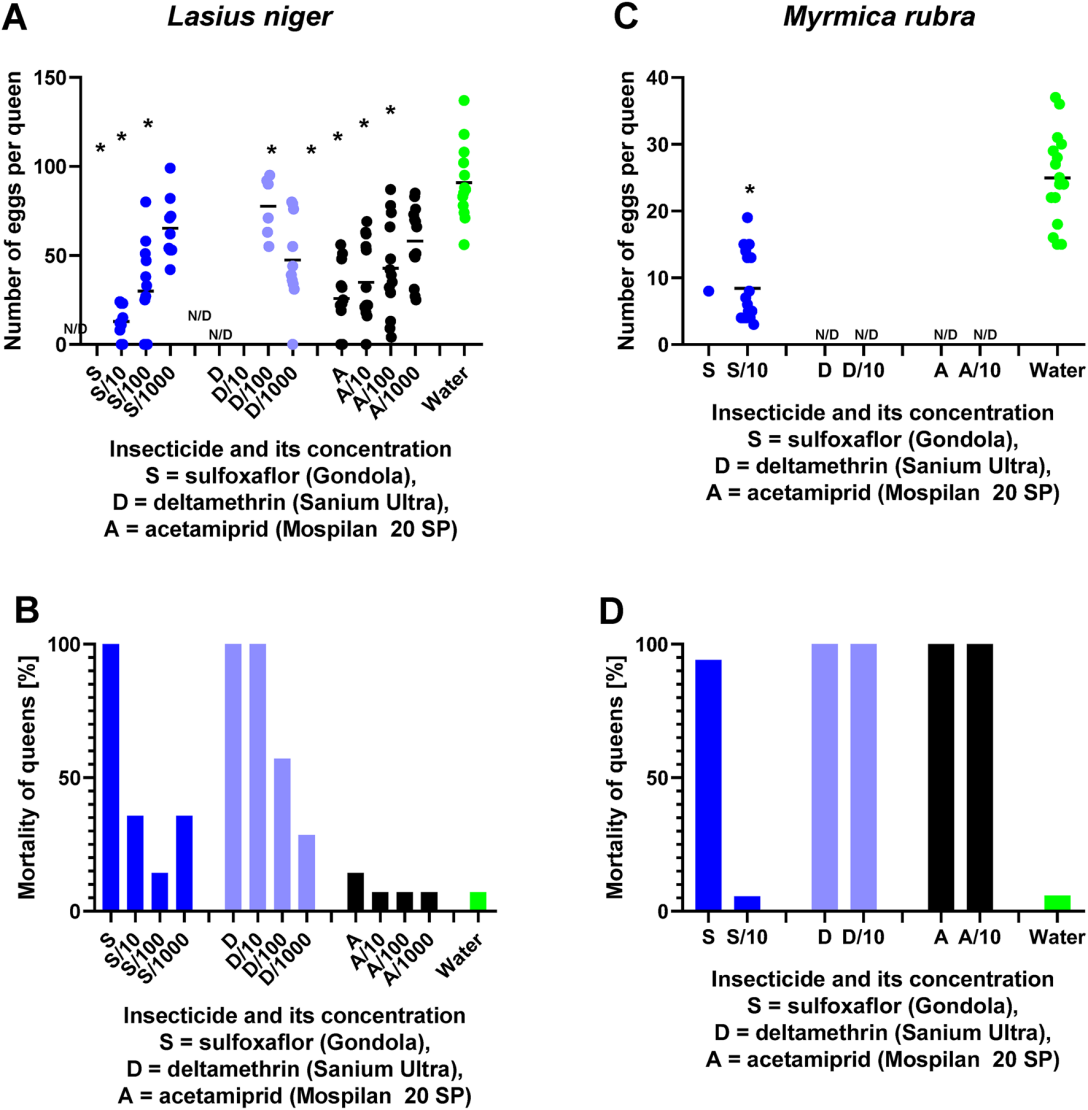
The effects of topical application (applied as a direct spray on the organism) of insecticide formulations on the the number of eggs produced per queen during the study period (**A**,**C**) and survival (**B**,**D**) of *L. niger* (**A**,**B**) and *M. rubra* (**C**,**D**) queens. The survival is quantified as the percentage of queens that survived the treatment and the follow-up period during the experiment. Only egg counts from queens that survived until the end of the experiment are shown. The maximum concentrations used: 1.25 g L^−1^ (acetamiprid, *A,* formulated as Mospilan), 0.6 g L^−1^ (sulfoxaflor, *S,* formulated as Gondola), and 0.875 g 1 L^−1^ (deltamethrin, *D,* formulated as Sanium Ultra). The concentrations lower by up to three orders of magnitude are indicated by fractions. Asterisks indicate significant numbers of eggs that significantly differed from the controls (Bonferroni post-test *p* < 0.05). Numbers of eggs are shown using individual datapoints, with short lines indicating the means. *ND* number of eggs was not defined because of 100% mortality of queens at the respective concentration.

All the tested Mospilan 20 SP concentrations significantly decreased the number of eggs produced by *L. niger* (one-way ANOVA F = 16.6, *p* < 0.001). The effects were concentration-dependent. While the control queens had the number of eggs at 90.9 ± 5.7 eggs per queen, the lowest concentration of Mospilan used (0.1% concentration) decreased the number of eggs to only 58.1 ± 5.5 eggs (Bonferroni post-test t = 3.7, *p* < 0.001). The number of eggs decreased to 42.8 ± 7.2 eggs at a 1% concentration of Mospilan, 34.9 ± 6.0 eggs at a 10% concentration of Mospilan, and only 25.8 ± 5.4 eggs at the recommended concentration of Mospilan (Fig. 2A,B). The concentration dependence can be expressed by a polynomial linear regression f = 58.2–0.34x (R^2^ 0.34, adjusted R^2^ 0.31, Shapiro–Wilk normality test P > 0.05, constant variance test P > 0.05).

In *M. rubra*, the recommended concentration of Mospilan and the treatment with 10% of the recommended concentration induced 100% lethality of the tested ant queens (Fig. 2C,D). When treated with these concentrations, *M. rubra* did not produce any eggs.

### Gondola

Gondola had more detrimental effects compared to the Mospilan. The recommended concentration of Gondola (0.6 g L^−1^) was lethal to all tested queens of *L. niger* (n = 14). The lower concentrations of Gondola were also associated with increased mortality (14–36% in each treatment). The Gondola-induced LD_50_ was 6.6% of the field-recommended dose (95% CI 1.6–27.9%; slope 0.699; intercept 4.367).

The surviving Gondola-treated queens of *L. niger* had significantly decreased the number of eggs (one-way ANOVA F = 32.5, *p* < 0.001). The effects were concentration-dependent. The lowest concentration of Gondola used (0.1% concentration) decreased the number of eggs to only 65.3 ± 5.5 eggs (Bonferroni post-test t = 3.7, *p* < 0.001). The number of eggs decreased to 29.9 ± 7.3 eggs at 1% concentration of Gondola, and only 12.9 ± 2.9 eggs at 10% of the recommended concentration of Gondola (Fig. 2A,B). The concentration dependence can be expressed by a polynomial linear regression f = 65.8–5.70x (R^2^ 0.38, adjusted R^2^ 0.36, Shapiro–Wilk normality test P > 0.05, constant variance test P > 0.05).

In *M. rubra*, the recommended concentration of Gondola induced 94% mortality. In contrast, 10% of the recommended concentration of Gondola induced only 5.6% mortality, similar to the mortality of queens subject to the control treatment (5.8%). The only queen of *M. rubra* that survived the treatment with recommended Gondola dose laid eight eggs. The *M. rubra* queens treated with 10% of the recommended concentration of Gondola also laid low numbers of eggs (8.4 ± 1.2, n = 17). The control, water-treated *M. rubra* queens laid 24.9 ± 1.7 eggs per queen (n = 17). The differences between the queens treated with 10% of the recommended concentration of Gondola and the control queens were statistically significant (*t*-test t = 7.84, *p* < 0.001) (Fig. 2C,D).

### Sanium Ultra

Sanium Ultra had the most detrimental effects among the three tested insecticides. The recommended concentration of Sanium Ultra (0.875 g 1 L^−1^) and 10% of the recommended concentration of Sanium Ultra were lethal to all tested queens of *L. niger* (n = 14 each). The lower concentrations of Sanium Ultra were also associated with increased mortality (29% and 14%, respectively). The Sanium Ultra-induced LD_50_ was 0.77% of the field-recommended dose (95% CI 0.21–2.78%; slope 0.824; intercept 5.123).

The surviving Sanium Ultra-treated queens of *L. niger* retained a relatively high number of eggs compared to the other two insecticides. Despite that, the declines in the number of eggs were significant (one-way ANOVA F = 11.1, *p* < 0.001). The lowest concentration of Sanium Ultra used (0.1% concentration) decreased the the number of eggs to only 47.4 ± 7.6 eggs (Bonferroni post-test t = 4.7, *p* < 0.001). However, the highest sublethal dose of Sanium Ultra (1% of the recommended concentration) did not induce a significant decrease in the number of eggs, and it remained at 77.7 ± 6.3 eggs per queen (Bonferroni post-test t = 1.2, *p* > 0.05) (Fig. 2A,B). The concentration dependence can be expressed by a polynomial inverse third-order regression f = 111.1 + (− 1121.1/x) + (1111/x^2^) + (− 100/x^3^) (R^2^ 1.00, adjusted R^2^ 1.00, Shapiro–Wilk normality test P > 0.05, constant variance test P < 0.01).

In *M. rubra*, recommended concentration of Sanium Ultra and the treatment with 10% of the recommended concentration induced 100% lethality of the tested ant queens (Fig. 2C,D). In contrast to other treatments, Sanium-Ultra-induced death was observed within an hour after the treatment.

## Discussion

All three tested insecticides caused severe lethal and sublethal concentration-dependent effects. The sublethal effects remained significant even when we decreased insecticide concentrations by three orders of magnitude compared to their recommended dosage. The decrease in concentrations by three orders of magnitude (compared to the concentrations recommended for foliar applications) was insufficient to avoid the sublethal effects of these insecticides. These concentrations caused severe declines in the number of eggs (and lethality at concentrations closer to the recommended ones). A higher number of eggs likely results in a larger workforce and a larger workforce is likely to result in a greater number of individuals of reproducing ant castes (drones and gynes)^59,60^. In social insects, reproductive success is determined by the number of drones and gynes, which successfully contribute to the foundation of new colonies^61,62^. Therefore, low doses of the tested insecticides can potentially decrease ant colonies' fitness substantially, and their use may lead to colony death. Massively occurring colony deaths adversely affect other organisms closely bound to ants. These include, for example, myrmecochorous plants, which seeds are dispersed by ant workers^63^. The susceptibility to agrochemicals varies among the ant species^64–66^.

The three studied groups of insecticides have a broad range of detrimental effects on ants. Regarding neonicotinoids, the previous studies reported acute lethal effects and cumulative toxicity in *Linepithema humile* (Mayr, 1868) (Hymenoptera: Formicidae)^67^. The extrapolation from acute to long-term effects is essential, particularly for the long-lived species, like the studied species *L. niger*, the queens of which have a lifespan of up to 30 years. Sublethal effects of neonicotinoids were studied but included the effects of imidacloprid, thiacloprid, and thiamethoxam^65,68–71^. The first of the mentioned studies reported that *L. humile* colonies produced significantly less brood when treated with sublethal concentrations of imidacloprid^65^. We found that queens had a lower number of eggs with increasing dose of insecticide, irrespective of the type of insecticide used. These findings are in line with Barbieri et al.^65^ who showed that *L. humile* produced fewer brood when treated with sublethal concentrations of the neonicotinoid imidacloprid. A lower egg-laying rate could also explain the results reported by Schläppi et al.^71^, who showed that despite thiamethoxam exposure having weak effects on the colony size after the first overwintering, it strongly affected the colony size after the second overwintering. As we show in the present study, exposure to the neonicotinoid insecticide acetamiprid was also directly related to the decline in the number of eggs (Fig. 2). Effects on the development of insects were previously shown for Mospilan in the more commonly studied groups of arthropods, like in the solitary bee *Osmia bicornis* (Linnaeus, 1758) (Hymenoptera: Megachilidae). Mospilan-treated larvae of *O. bicornis* have difficulty emerging when fed with Mospilan-contaminated pollen^72^.

Pyrethroids are known to dysregulate the function of the ovary, particularly the development of follicles and reproductive hormone levels^73^. The studies on ants mainly focus on the lethal effects of pyrethroids [e.g., Refs.^74,75^]. Sublethal concentrations of lambda-cyhalothrin delay the growth of *M. rubra* larvae and reduce the adult body mass of males^76^. In the present study, we show that severe sublethal effects of pyrethroids must be considered, as they were still detectable when we decreased the working concentrations by three orders of magnitude (Fig. 2). Sublethal doses of deltamethrin (the active compound of Sanium Ultra) reduce the fertility of honeybees and parasitoid wasps^77,78^, impair larval development in honeybees^79^ and inhibit molting processes in the fly *Stomoxys calcitrans* (Linnaeus, 1758) (Diptera: Muscidae)^80^.

The third group of insecticides, sulfilimines, is used mainly against sap-feeding insects. Sulfoxaflor is, so far, the only frequently applied sulfilimine insecticide^81^. These authors concluded that sulfoxaflor is much less active against other insects, including *Diabrotica undecimpunctata howardi* Barber, 1947 and *Leptinotarsus decemlineata* Say, 1824 (both Coleoptera: Chrysomelidae), than neonicotinoids. Sulfoxaflor was expected to replace neonicotinoids in areas of their ban^32^. However, note that on April 7, 2022, the European Commission announced an upcoming ban on the outdoor use of sulfoxaflor in the European Union^82^ because of the evidence-based data on its adverse effects on pollinators and biodiversity^83^. It degrades more quickly than the neonicotinoids but still persists in the nectar and pollen for at least 11 days, which is the maximum tested interval^84,85^. However, severe mortality, decreased food consumption, and reduced interspecific aggressiveness were reported in *S. invicta* treated with sulfoxaflor at 1 μg mL^−1^ and 2 μg mL^−1^ *p.o.*^34^. Similarly, mortality, decreased locomotion, and altered interactions were reported in *Tetramorium caespitum* (Linnaeus, 1758) (Hymenoptera: Formicidae) treated with sulfoxaflor at concentrations from 1 mg mL^−1^ to 50 mg mL^−1^ *p.o.*^35^. In the present study, we found that the field-recommended concentrations of sulfoxaflor and even concentrations lower by three orders of magnitude are sufficient to induce severe declines in the number of eggs produced by ant queens (Fig. 2). In honeybees, sublethal doses of sulfoxaflor disrupt the development of larvae and lead to metamorphosis to adults failure^86^. Post-spray field exposure of 5 ng g^−1^ decreased the number of reproductive offspring in bumblebees^30^ and reduced the number of bumblebee eggs and larvae^31^.

The presence and abundance of both studied ant species strongly influence the populations of other arthropods: the density of Collembola, Hemiptera (non-tended by ants), spiders, and hymenopteran parasitoid *Blacus* spp. (Hymenoptera: Braconidae) increased in plots with *L. niger* and/or *M. rubra* compared to plots without ants, but the effect varied with ant species, the duration of the experiment, and ant abundance^87^. Thus, the insecticide-driven reduction of ant fitness may project beyond ants. These effects may extend to species that may not be sensitive to the respective agrochemical populations. *Lasius niger* is associated with a broader spectrum of myrmecophilous lycaenid butterflies concerning the two studied species. However, caterpillars of obligately myrmecophilous and strongly endangered *Phengaris* (Lepidoptera: Lycaenidae) species develop in *Myrmica* (including *M. rubra*) colonies^88^. *Lasius niger* changes the chemical properties of soil and vegetation surrounding their nests differently and to a larger extent than the *M. rubra*^89,90^. *Myrmica rubra* is more effective in dispersing seeds of myrmecochorous plants^91^. They also differ in prey specialization, as *L. niger* focuses predominantly on aphid honeydew, whereas *M. rubra* is considered rather predatory^92^.

A major limitation of the present study consists of the use of only two study species. Further research should elucidate, whether the observed differences between the two studied species were species-specific, or whether they were indeed related to their different colony founding strategies. Queens of *L. niger* do not forage and utilize their wing musculature until the first workers emerge, representing characteristic claustral colony founding^93^. In contrast, the wing musculature of queens of *M. rubra* is less developed. Thus, the *M. rubra* queens must hunt to feed themselves and their larvae, representing a characteristic semiclaustral colony founding mode^21^. Multiple species within the claustral and semiclaustral colony founding categories need to be tested to provide a definitive answer.

As the studied insecticides have detrimental effects on the survival and the number of eggs of both studied ant species, safer alternatives are needed. This also calls for improving approval procedures for these insecticides to avoid the repeatedly happening situation when a well-characterized insecticide with known adverse effects is replaced with its more recent derivative, for which the knowledge of non-target effects is limited. This applies even to bioinsecticides; all newly developed compounds must be thoroughly tested before their approval as they also may be toxic to organisms and the environment^94^. In this regard, it is essential to note that ants are not considered soil-dwelling organisms and, thus, are not subject to current EFSA and OECD risk assessment schemes^95,96^. Another issue is the missing data on novel formulations of already approved compounds. The formulations with improved insecticidal properties may have a prolonged half-life and increased bioavailability, which can also be associated with increased toxicity^97,98^. Nanoformulations of the tested compounds were already published^99–101^. Therefore, their effects on ants and other organisms must be thoroughly tested. The extent of the detrimental effects of the examined insecticides on the two tested common ant species was unexpected. It may partly explain the recent declines in insect diversity in agricultural landscapes. Further research should extend the study to the field conditions and consider insecticides' effects that could be related to the eusocial aspect of the studied species. The approvals of newly released agrochemicals should not be allowed unless they are tested for adverse effects using robust risk assessment schemes. These schemes must involve representatives of organisms affected by related chemical compounds. In the case of newly released neonicotinoid formulations, these organisms would include not only honey bees and bumblebees (these are represented now) but also ants.

## Data Availability

All data generated or analyzed during this study are included in this published article.

## References

[1] Thompson HM, Maus C (2007). The relevance of sublethal effects in honey bee testing for pesticide risk assessment. Pest Manag. Sci..

[2] Rose RI (2006). Tier-based testing for effects of proteinaceous insecticidal plant-incorporated protectants on non-target arthropods in the context of regulátory risk assessments. IOBC WPRS Bull..

[3] Schläppi D, Stroeymeyt N, Neumann P (2021). Unintentional effects of neonicotinoids in ants (Hymenoptera: Formicidae). Myrmecol. News.

[4] Ricupero M, Desneux N, Zappalà L, Biondi A (2020). Target and non-target impact of systemic insecticides on a polyphagous aphid pest and its parazitoid. Chemosphere.

[5] Gontijo PC, Neto DOA, Oliveira RL, Michaud JP, Carvalho GA (2018). Non-target impacts of soybean insecticidal seed treatments on the life history and behavior of *Podisus nigrispinus*, a predator of fall armyworm. Chemosphere.

[6] Main AR, Webb EB, Goyne KW, Mengel D (2018). Neonicotinoid insecticides negatively affect performance measures of non-target terrestrial arthropods: A meta-analysis. Ecol. Appl..

[7] Desneux N, Decourtye A, Delpuech J-M (2007). The sublethal effects of pesticides on beneficial arthropods. Annu. Rev. Entomol..

[8] Evans AN, Llanos JEM, Kunin WE, Evison SEF (2018). Indirect effects of agricultural pesticide use on parasite prevalence in wild pollinators. Agric. Ecosyst. Environ..

[9] Korenko S, Saska P, Kysilková K, Řezáč M, Heneberg P (2019). Prey contaminated with neonicotinoids induces feeding deterrent behavior of a common farmland spider. Sci. Rep..

[10] Stanley DA, Garratt MP, Wickens JB, Wickens VJ, Potts SG, Raine NE (2015). Neonicotinoid pesticide exposure impairs crop pollination services provided by bumblebees. Nature.

[11] Chagnon M, Kreutzweiser D, Mitchell EA, Morrissey CA, Noome DA, Van der Sluijs JP (2015). Risks of large-scale use of systemic insecticides to ecosystem functioning and services. Env. Sci. Poll. Res..

[12] Del Toro I, Ribbons R, Pelini S (2012). The little things that run the world revisited: A review of anti-mediated ecosystem services and disservices (Hymenoptera: Formicidae). Myrmecol. News.

[13] Vandermeer J, Perfecto I, Ibarra-Núñez G, Philpott S, Garcia-Ballinas JA (2002). Ants (*Azteca* sp.) as potential biological control agents in organic chade coffee production in Southern Chiapas, Mexico. Agrofor. Syst..

[14] Chailleux A, Stirnemann A, Leyes J, Deletre E (2019). Manipulating natural enemy behavior to improve biological control: Attractants and repellents of a weaver ant. Entomol. Gen..

[15] Frizzo TL, Souza LM, Sujii ER, Togni PH (2020). Ants provide biological control on tropical organic farms influenced by local and landscape factors. Biol. Control.

[16] Hölldobler B, Wilson EO (1990). The Ants.

[17] Frouz J, Jílková V (2008). The effect of ants on soil properties and processes (Hymenoptera: Formicidae). Myrmecol. News.

[18] Schläppi D (2021). Varying impact of neonicotinoid insecticide and acute bee paralysis virus across castes and colonies of black garden ants, *Lasius niger* (Hymenoptera: Formicidae). Sci. Rep..

[19] Song Y (2019). Research progress on bouncing behaviour and control technology of pesticide droplets at plant leaf surface. Chin. J. Pesticide Sci..

[20] Sluijs, J. van der P. *et al*. Neonicotinoids, bee disorders and the sustainability of pollinator services. *Curr. Opin. Environ. Sust.* **5**, 293–305 (2013).

[21] Seifert B (2018). The Ants of Central and North Europe.

[22] Simon-Delso N (2015). Systemic insecticides (neonicotinoids and fipronil): Trends, uses, mode of action and metabolites. Env. Sci. Poll. Res..

[23] Epstein Y, Chapron G, Verheggen F (2021). EU Court to rule on banned pesticide use. Science.

[24] Jactel H (2019). Alternatives to neonicotinoids. Environ. Int..

[25] Azpiazu C (2021). Toxicity of the insecticide sulfoxaflor alone and in the combination with the fungicide fluxapyroxad in three bee species. Sci. Rep..

[26] Naharashi T, Frey JM, Ginsburg KS, Roy ML (1992). Sodium and GABA-activated channels as the targets of pyrethroids and cyclodienes. Toxicol. Lett..

[27] Davies TGE, Field LM, Usherwood PNR, Williamson MS (2007). DDT, pyrethrins, pyrethroids and insect sodium channels. IUBMB Life.

[28] Desneux N (2005). *Diaeretiella rapae* limits *Myzus persicae* populations after applications of deltamethrin in oilseed rape. J. Econ. Entomol..

[29] Longhurst C (2013). Cross-resistance relationships of the sulfoximine insecticide sulfoxaflor with neonicotinoids and other insecticides in the whiteflies *Bemisia tabaci* and *Trialeurodes vaporariorum*. Pest Manag. Sci..

[30] Siviter H, Brown MJF, Leadbeater E (2018). Sulfoxaflor exposure reduces bumblebee reproductive success. Nature.

[31] Siviter H, Homer J, Brown MJF, Leadbeater E (2020). Sulfoxaflor exposure reduces egg laying in bumblebees *Bombus terrestris*. J. Appl. Ecol..

[32] Siviter H, Muth F (2020). Do novel insecticides pose a threat to beneficial insects?. Proc. R. Soc. B.

[33] Siviter H (2019). No evidence for negative impacts of acute sulfoxaflor exposure on bee olfactory conditioning or working memory. PeerJ.

[34] Pan F, Lu Y, Wang L (2017). Toxicity and sublethal effects of sulfoxaflor on the red imported fire ant, *Solenopsis invicta*. Ecotoxicol. Environ. Saf..

[35] Frankel TE, Frankel JS (2021). Sulfoxaflor causes mortality, decreased locomotion, and altered interactions in pavement ants (*Tetramorium caespitum*). J. Environ. Sci. Health B.

[36] Erickson BE (2023). EPA to reconsider sulfoxaflor`s risk. C&EN.

[37] Trompiz, G. French court suspends two Dow pesticides over potential harm to bees. Reuters, 24-Nov-2017. https://www.reuters.com/article/us-france-pesticides-idUSKBN1DO1M9 Accessed 12 July 2023.

[38] Gill P (2021). Assessment of neonicotinoid insecticide adetamiprid LC50 against earthworm (*Eisenia fetida* L.). Environ. Ecol..

[39] Song Y, Kai J, Song X, Zhang W, Li L (2015). Long-term effects of deltamethrin and fenvalerante in soil. J. Hazard. Mat..

[40] Fang S (2018). Lethal toxicity and sublethal metabolic interference effects of sulfoxaflor on the earthworm (*Eisenia fetida*). J. Agric. Food Chem..

[41] Potts J, Cross P, MacDonald A, Jones D (2019). Acetamiprid transport and mobility within UK agricultural soils—A comparison of commercial mixtures under different soil organic matter treatments. Geophys. Res. Abstr..

[42] Zhang L, Khan SU, Akhtar MH, Ivarson KC (1984). Persistence, degradation, and distribution of deltamethrin in an organic soil under laboratory conditions. J. Agric. Food Chem..

[43] Vig K, Singh DK, Agarwal HC, Dhawan AK, Dureja P (2001). Insecticide residues in cotton crop soil. J. Environ. Sci. Health B.

[44] USEPA/OPPTS. Pesticide Fact Sheet: Sulfoxaflor. EPA, Washington, DC. https://www.epa.gov/pesticides/factsheets/index.htm (2013).

[45] NCBI. PubChem Compound Summary for CID 16723172, Sulfoxaflor. NCBI, Bethesda. https://pubchem.ncbi.nlm.nih.gov/compound/Sulfoxaflor (2022).

[46] Peck SL, McQuaid B, Campbell CL (1998). Using ant species (Hymenoptera: Formicidae) as a biological indicator of agroecosystem condition. Environ. Entomol..

[47] Rodríguez, E., Peña, A., Raya, A. J. S., Campos, M. (2003). Evaluation of the effect on arthropod populations by using deltamethrin to control *Phloeotribus scarabaeoides* Bern. (Coleoptera: Scolytidae) in olive orchards. *Chemosphere* **52**, 127–134. 10.1016/S0045-6535(03)00184-X 12729695

[48] Wetterer JK, Radchenko AG (2011). Worldwide spread of the ruby ant, *Myrmica rubra* (Hymenoptera: Formicidae). Myrmecol. News.

[49] Schär S (2018). Do Holarctic ant species exist? Trans-Beringian dispersal and homoplasy in the Formicidae. J. Biogeogr..

[50] Schär S (2022). Integrative taxonomy reveals cryptic diversity in North American *Lasius* ants, and an overlooked introduced species. Sci. Rep..

[51] Rasse P, Deneubourg JL (2001). Dynamics of nest excavation and nest size regulation of *Lasius niger* (Hymenoptera: Formicidae). J. Insect Behav..

[52] Radchenko AG, Elmes GW (2010). *Myrmica* ants of the old world. Fauna Mundi.

[53] Anonymus. MOSPILAN 20 SP. https://www.agromanual.cz/download/pdf_etiketa/e_mospilan_20_sp.pdf (2021).

[54] Gupta S, Gajbhiye VT (2007). Persistence of acetamiprid in soil. Bull. Environ. Toxicol..

[55] Anonymus. Sanium Ultra. https://www.prohopo.cz/userfiles/files/1240047_P%C5%99%C3%ADbalov%C3%BD%20let%C3%A1k%20(SBM%20Life%20Science)%20Sanium%20Ultra.pdf (2022).

[56] Selim H, Zhu H (2002). Retention and mobility of deltamethrin in soils: 2. Transport 1. Soil Sci..

[57] Anonymus. Gondola. https://www.agrofert.cz/sites/default/files/downloads/gondola_0.pdf (2022).

[58] Finney DJ (1971). Probit analysis. J. Pharm. Sci..

[59] Dickinson JL, Hatchwell B, Koenig WD, Dickinson JL (2004). Fitness consequences of helping. Ecology and Evolution of Cooperative Breeding in Birds.

[60] Bernasconi G, Strassmann JE (1999). Cooperation among unrelated individuals: The ant foundress case. Trends Ecol. Evol..

[61] Lopez-Vaamonde C (2009). Lifetime reproductive success and longevity of queens in an annual social insect. J. Evol. Biol..

[62] Keller L (1993). The assessment of reproductive success of queens in ants and other social insects. Oikos.

[63] Gammans N, Bullock JM, Schönrogge K (2005). Ant benefits in a seed dispersal mutualism. Plant Anim. Interact..

[64] Gibson RL, Scott JG (1989). Comparative toxicity of fourteen insecticides to two species of carpenter ants (Hymenoptera:Formicidae). J. Econ. Entomol..

[65] Barbieri RF, Lester PJ, Miller AS, Ryan KG (2013). A neurotoxic pesticide changes the outcome of aggressive interactions between native and invasive ants. Proc. R. Soc. B.

[66] Heneberg P, Svoboda J, Pech P (2021). Claustral colony founding does not prevent sensitivity to the detrimental effects of azole fungicides on the fecundity of ants. J. Environ. Manag..

[67] Rust MK, Reierson DA, Klotz JH (2004). Delayed toxicity as a critical factor in the efficacy of aqueous baits for controlling Argentine ants (Hymenoptera: Formicidae). J. Econ. Entomol..

[68] Wang L, Zeng L, Chen J (2015). Impact of imidacloprid on new queens of imported fire ants, *Solenopsis invicta* (Hymenoptera: Formicidae). Sci. Rep..

[69] Thiel S, Köhler H-R (2016). A sublethal imidacloprid concentration alters foraging and competition behaviour of ants. Ecotoxicology.

[70] Jung J-K, Jung C, Koh S-H (2018). Lethal and sublethal effects of thiacloprid on non-target carpenter ant, *Camponotus japonicas* Mayr (Hymenoptera: Formicidae). J. Asia-Pac. Entomol..

[71] Schläppi D, Kettler N, Straub L, Glauser G, Neumann P (2020). Long-term effects of neonicotinoid insecticides on ants. Commun. Biol..

[72] Mokkapati JS, Bednarska AJ, Laskowski R (2021). The development of the solitary bee *Osmia bicornis* is affected by some insecticide agrochemicals at environmentally relevant concentrations. Sci. Total Environ..

[73] Jurewicz J (2020). Exposure to pyrethroid pesticides and ovarian reserve. Environ. Int..

[74] Soeprono AM, Rust MK (2004). Effect of delayed toxicity of chemical barriers to control Argentine ants (Hymenoptera: Formicidae). J. Econ. Entomol..

[75] Sakamoto H, Goka K (2021). Acute toxicity of typical ant control agents to the red imported fire ant, *Solenopsis invicta* (Hymenoptera: Formicidae). Appl. Entomol. Zool..

[76] Müller T, Gesing MA, Segeler M, Müller C (2019). Sublethal insecticide exposure of an herbivore alters the response of its predator. Environ. Pollut..

[77] Dai PL (2010). Effects of sublethal concentrations of bifenthrin and deltamethrin on fecundity, growth, and development of the honeybee *Apis mellifera ligustica*. Environ. Toxicol. Chem..

[78] Teder T, Knapp M (2019). Sublethal effects enhance detrimental impact of insecticides on non-target organisms: A quantitative synthesis in parasitoids. Chemosphere.

[79] Yang Y (2020). Acute and chronic toxicity of acetamiprid, carbaryl, cypermethrin and deltamethrin to *Apis mellifera* larvae reared *in vitro*. Pest Manag. Sci..

[80] Reissert-Oppermann S, Bauer B, Steuber S, Clausen PH (2019). Insecticide resistence in stable flies (*Stomoxys calcitrans*) on dairy farms in Germany. Parasitol. Res..

[81] Babcock JM (2011). Biological characterization of sulfoxaflor, a novel insecticide. Pest Manag. Sci..

[82] Directorate-General for Health and Food Safety. Sulfoxaflor: Commission restricts the use of harmful pesticide for pollinators. https://food.ec.europa.eu/news/sulfoxaflor-commission-restricts-use-harmful-pesticide-pollinators-2022-04-07_en Accessed 3 August 2023.

[83] EFSA, *et al*. Peer review of the pesticide risk assessment for the active substance sulfoxaflor in light of confirmatory data submitted. *EFSA J.* **17**, 5633 (2019). 10.2903/j.efsa.2019.5633 PMC7009138 32626258

[84] EPA. *Addendum to the environmental fate and ecological risk assessment for sulfoxaflor registration.* (Environmental Protection Agency, Washington, 2016)

[85] EPA. *Decision memorandum supporting the registration decision for new uses of the active ingredient sulfoxaflor on alfalfa, cacao, citrus, corn, cotton, cucurbits, grains, pineapple, sorghum, soybeans, strawberries and tree plantations*. (Environmental Protection Agency, Washington, 2019)

[86] Li J (2021). Sublethal effects of Isoclast™ Active (50% sulfoxaflor water dispersible granules) on larval and adult worker honey bees (*Apis mellifera* L.). Ecotoxicol. Environ. Saf..

[87] Sanders D, van Veen FJF (2011). Ecosystem engineering and predation: The multi-trophic impact of two ant species. J. Anim. Ecol..

[88] Fiedler K (2006). Ant-associates of Palaearctic lycaenid butterfly larvae (Hymenoptera: Formicidae; Lepidoptera: Lycaenidae)—A review. Myrmecol. News.

[89] Petal J (1980). Ant populations, their regulation and effect on soil in meadows. Ekol. Pol..

[90] Petal J, Kusinska A (1994). Fractional composition of organic matter in the soil of anthills and of the environment of meadows. Pedobiol..

[91] Servigne P, Detrain C (2008). Ant-seed interactions: Combined effects of ant and plant species on seed removal patterns. Insectes Soc..

[92] Fiedler K, Kuhlmann F, Schlick-Steiner BC, Steiner FM, Gebauer G (2007). Stable N-isotope signatures of central European ants—Assessing positions in a trophic gradient. Insectes Soc..

[93] Keller L, Passera L (1989). Size and fat content of gynes in relation to the mode of colony founding in ants (Hymenoptera: Formicidae). Oecologia.

[94] Lamichhane JR (2018). Thirteen decades of antimicrobial copper compounds applied in agriculture. A review. Agron. Sustain. Dev..

[95] EFSA Ppr Panel (2017). Scientific opinion addressing the state of the science on risk assessment of plant protection products for in-soil organisms. EFSA J..

[96] OECD. OECD Guidelines for the Testing of Chemicals, Section 2 - Effects on Biotic Systems. 10.1787/20745761 (2019).

[97] Fojtová D (2019). Nanoformulations can significantly affect pesticide degradation and uptake by earthworms and plants. Environ. Chem..

[98] Xu Z (2022). Environmental risks and the potential benefits of nanopesticides: A review. Environ. Chem. Lett..

[99] Padmavathi P, Vasundhara N, Kovvuri S, Venugopal N (2020). Synthesis and characterization of nano-acetamiprid-new plant safeguard material. Am. J. Anal. Chem..

[100] Ebadollahi A (2022). Nanoencapsulation of acetamiprid by sodium alginate and polyethylene glycol enhanced its insecticidal efficiency. Nanomaterials.

[101] Khalifa AG (2022). Deltamethrin and its nanoformulations induce behavioral alteration and toxicity in rat brain through oxidative stress and JAK2/STAT3 signaling pathway. Toxics.

